# Automated analyser for monitoring trace amounts of volatile chloro-organic compounds in recirculated industrial water

**DOI:** 10.1155/S1463924602000020

**Published:** 2002

**Authors:** Wacław Janicki, Wojciech Chrzanowski, Andrzej Wasik, Elżbieta Przyk, Jacek Namieśnik

**Affiliations:** Department of Analytical Chemistry Chemical Faculty Technical University of Gdańsk 11/12 Narutowicza Street Gdańsk 80-952 Poland

## Abstract

An automated analyser of volatile chloro-organic compounds in water was constructed and tested using standard mixtures of dichloromethane and dichloroethane. It was based on continuous, countercurrent gas stripping of the liquid sample followed by periodic trapping of the analytes on two traps alternately connected to the bubbler outlet, and thermal desorption. When one trap performed adsorption, the other underwent desorption and cooling. Analytes were detected by an ECD detector. Integration, calibration, calculations and overall operating cycle control was performed by a microcomputer. The instrument guarantees a 0.02 ppm Cl (w/w) detection limit, a 0—2 ppm detection range and 2 months of autonomous operation. Results are reported every 13 min.


Journal of Automated Methods & Management in Chemistry
Vol. 24, No. 1 (January–February 2002) pp. 9–16

Taylor & Francis

Taylor & Francis Group

### Automated analyser for monitoring trace amounts of volatile chloro-organic compounds in recirculated industrial water

**Wacław Janicki, Wojciech Chrzanowski*, Andrzej Wasik, Elżbieta Przyk and Jacek Namieśnik**

*Department of Analytical Chemistry, Chemical Faculty, Technical University of Gdańsk, 11/12 Narutowicza Street, 80-952 Gdańsk, Poland*

*An automated analyser of volatile chloro-organic compounds in water was constructed and tested using standard mixtures of dichloromethane and dichloroethane. It was based on continuous, countercurrent gas stripping of the liquid sample followed by periodic trapping of the analytes on two traps alternately connected to the bubbler outlet, and thermal desorption. When one trap performed adsorption, the other underwent desorption and cooling. Analytes were detected by an ECD detector. Integration, calibration, calculations and overall operating cycle control was performed by a microcomputer. The instrument guarantees a 0.02 ppm Cl (w/w) detection limit, a 0–2 ppm detection range and 2 months of autonomous operation. Results are reported every 13 min.*

### Introduction

In the chemical industry, which is highly water consuming, recirculation of the costly de-ionized water used in both steam generation and technological processes is usually required. In oil refineries, there are some processes using a mixture of dichloromethane and dichloroethane (DIMA), which is cooled with water. Any leak in these installations would lead to contamination of technological water with the compounds and their subsequent hydrolysis under high pressure and temperature conditions of the boilers. Hydrochloric acid produced is a major corrosion agent and its presence should be avoided or, at least, a suitable warning system should be applied for continuous monitoring of chlorine contents in recirculated water and condensate. The Gdańsk Refinery, after a year lasting worldwide searches for such instruments, concluded that they were not yet commercially available and ordered construction of the instrument at the TUG. The specification included the following requirements.

- Detection limit: 0.02 ppm Cl (w/w).
- Measuring range: 0–2 ppm Cl (w/w).
- Precision: not worse than 5% relative.
- Warning time: maximum 20 min.
- Period of autonomous operation: minimum of 2 months.
- Transmission of data to the main control room of the plant.

*To whom correspondence should be addressed. e-mail: wojtek@altis.chem.pg.gda.pl

The instrument thus designed and described in this paper was designated model DCH-01 automatic analyser of volatile chloro-organic compounds in water.

### Operating principle

The instrument utilizes the purge and trap principle [1–5], which is particularly suitable for the isolation of volatile chloro-organic compounds from water [3, 6, 7], and, hence, operates in a periodic mode. It was decided to utilize not the total stripping but rather a dynamic one, when the liquid sample and the stripping gas, both at constant flow rates, come into contact with each other in a countercurrent purging bubbler, where the volatile chloro-organic compounds are divided between the two phases with their respective partition coefficients [6] kept constant by maintaining the temperature at $25 \pm 2\,^{\circ}C$. This arrangement permitted the shortening of the time needed for completion of a single analysis cycle, as the stripping time is limited to such which is necessary to liberate from an analysed water sample a detectable amount of analytes without a provision of their removal from the sample being complete.

A schematic diagram of the instrument is shown in figure 1. Water to be analysed flows to the sample tank (ST) from an industrial recirculation system. The system for cooling the sample, which is chiefly warm condensate, along with a suitable pressure reduction system, must be provided by the customer. Three-way electromagnetic valves (V6, V7) permit switching between the sample tank and the standard solution tank (StT), the latter being used when the calibration is performed. Pump (P), model R408.1 (SeRa, Germany, maximum output 2.4 $l\,h^{-1}$, adjustable, maximum operating pressure 1 MPa) delivers the sample to the purging bubbler, where stripping occurs. The depleted sample flows to the dump through the system of valves, two of them (V2, V1) permit the maintenance of a constant level of water in the bubbler, while the other two (V8, V9) serve for the adjustment of an analysed water flow rate. Nitrogen of high purity (99.999%) from a cylinder, after pressure reduction, flows via another three-way electromagnetic valve (V3) either to the bubbler or to the traps (T1, T2). On its way to the bubbler, it passes through a pressure/flow regulator (P/FR) and an electronic manometer (MM). After the bubbler, it goes through a preliminary drier (Dr1), in which small droplets of water are removed and through the main drier (Dr2). The latter is of permeation type, utilizing a Nafion® tube allowing water vapour permeation through its walls to the water-retaining space, while keeping the analytes in the gas stream. The water retaining space is filled with a 5A

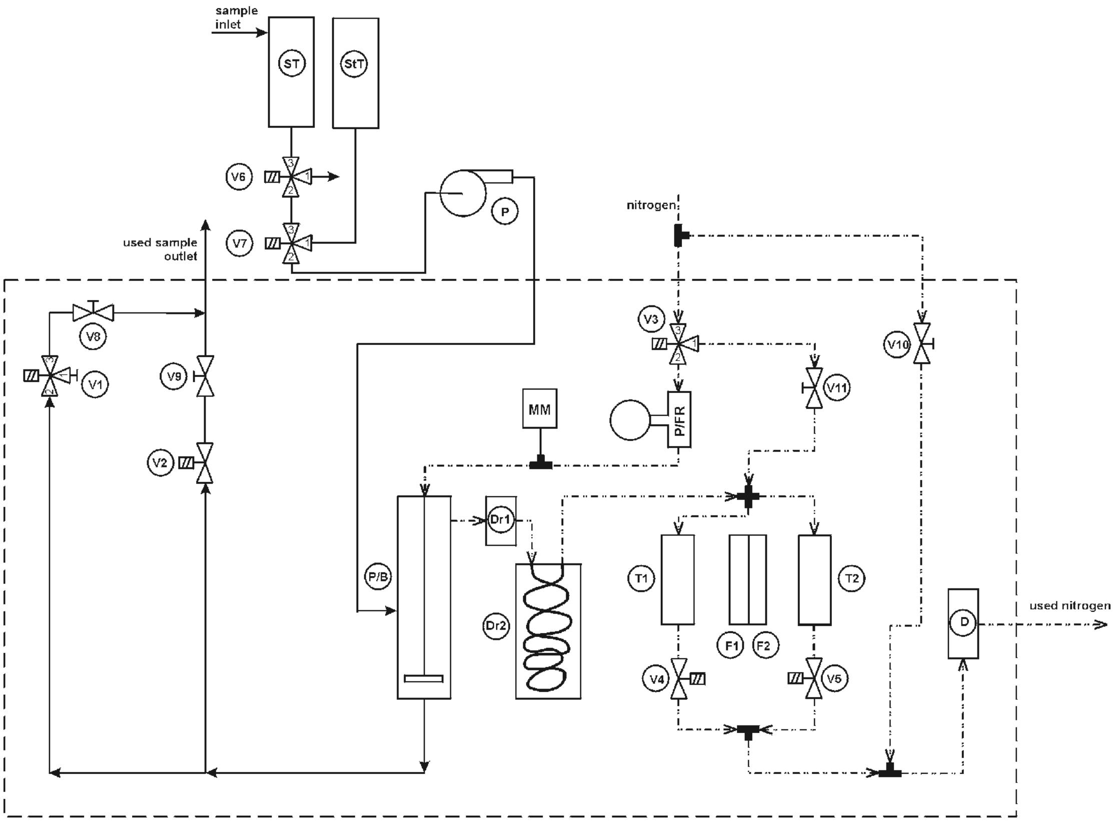

*Figure 1. Pneumatic/hydraulic system of a model DCH-01 automatic analyser of volatile chloro-organic compounds in water: ST, water sample tank; StT, standard solution tank; P, pump; P/B, purging bubbler; Dr1, droplet remover; Dr2, gas sample drying system; T1, T2, traps (Tenax TA® sorbent beds); F1, F2, cooling fans; D, detector (ECD Model 850); P/FR, pressure/flow regulator; MM, electronic manometer; V1 (3w, elm), main valve controlling the level of water in the bubbler; V2 (2w, elm), emergency bubbler draining valve; V3 (elm, 3w), main nitrogen manifold valve redirecting gas either to the bubbler or to the traps; V4 (2w, elm), T1 opening valve; V5 (2w, elm), T2 opening valve; V6 (3w, elm), main sample delivering valve; V7 (3w, elm), sample/standard solution switching valve; V8–11 (needle valves, manual), flow adjustment valves for: normal sample flow, emergency sample removal from the bubbler, make-up gas flow and trap desorption gas flow, respectively (2w, two-way valve; 3w, three-way valve; elm, electromagnetic valve). Solid lines represent the sample (water) transporting tubing; a dash-double dot line represents a gas transport tubing. Elements shown within a dashed line rectangle are placed within the instrument housing.*

molecular sieve ($\sim$200 g). Dry nitrogen, carrying the analytes, enters one of two traps (T1, T2), which are tubes filled with beds of about 0.21 g Tenax TA®, selected on the basis of earlier experience [3], each. Switching electromagnetic valves (V4, V5) permit the direction of the gas stream on one of the two traps. Use of two traps was also chosen to shorten the time per single analysis cycle. The analytes are retained on a cool ($< 38\,^{\circ}$C) sorbent bed. After completion of the stripping/sorption part of an analysis cycle, the stream of nitrogen is redirected by valve V3 directly to the traps and the currently working trap is heated to 250 °C (each trap is equipped with a 100-W heater). Nitrogen carries the concentrated 'band' of thermally desorbed analytes to the detector (D) after joining the make-up stream. The electron capture detector is a model 850 from CE Instruments (ThermoQuest, Rodano, Italy). The flow rate of the working stream of nitrogen may be adjusted by means of valve V12 and that of the make-up stream — by means of valve V11. Both traps are also equipped with fans (F1, F2) permitting their rapid cooling once the desorption is complete. Immediately after cooling of T1 begins, another stripping cycle starts with trapping the analytes on trap T2. The whole concept of dual trap operation is illustrated in figure 2, where states of key control elements (valves V3–5; heaters of traps T1 and T2; fans F1 and F2) are shown in time. It may be seen that a complete cycle for a single trap lasts 13 min, while the result (from both traps) is obtained in every 8 min.

All connections are made of stainless steel tubing (1/16 inch o.d.). The need of this material was duly proven during tests.

### Automation

It is obvious that unattended operation of the above-described instrument may be achieved exclusively by employment of a suitably sophisticated control system. A most general concept of this system is shown in figure 3. It was housed in a separate compartment of the instrument housing (figure 4). The computer employed is IBM compatible, assembled in the instrument housing, with an LCD display and mini-keyboard (collapsible) mounted in the door. It also includes an Intel®

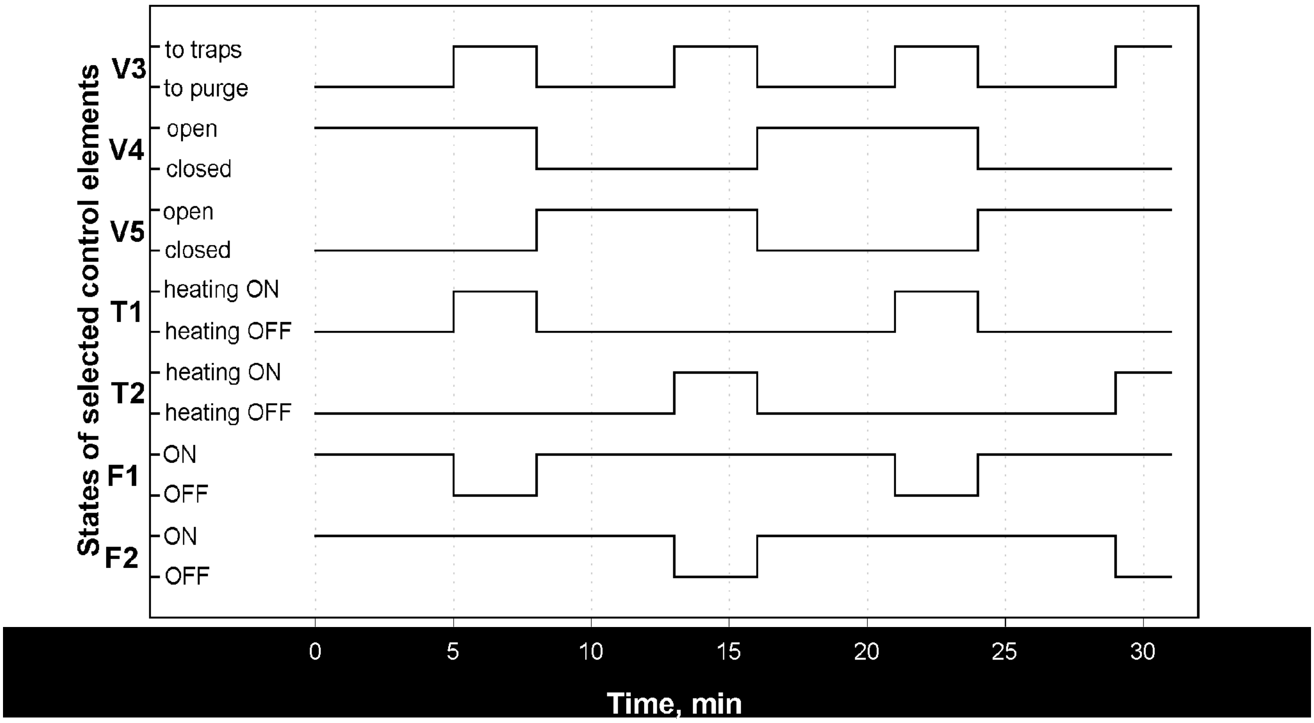

*Figure 2. Operation cycle of the instrument, states of key control elements in time: T1, T2, heater of traps; F1, F2, cooling fans; V3, main nitrogen manifold valve redirecting gas either to the bubbler or to the traps; V4, T1 opening valve; V5, T2 opening valve.*

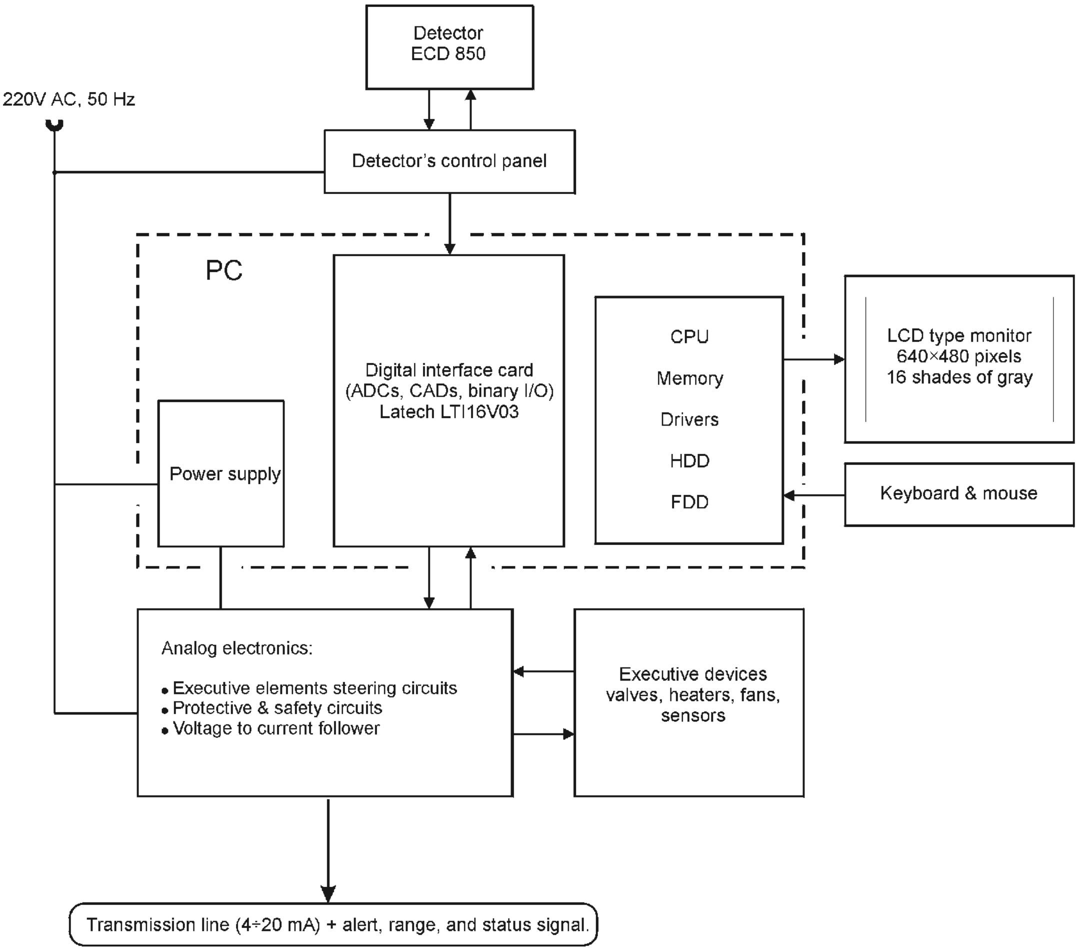

*Figure 3. Instrument automatic control system.*

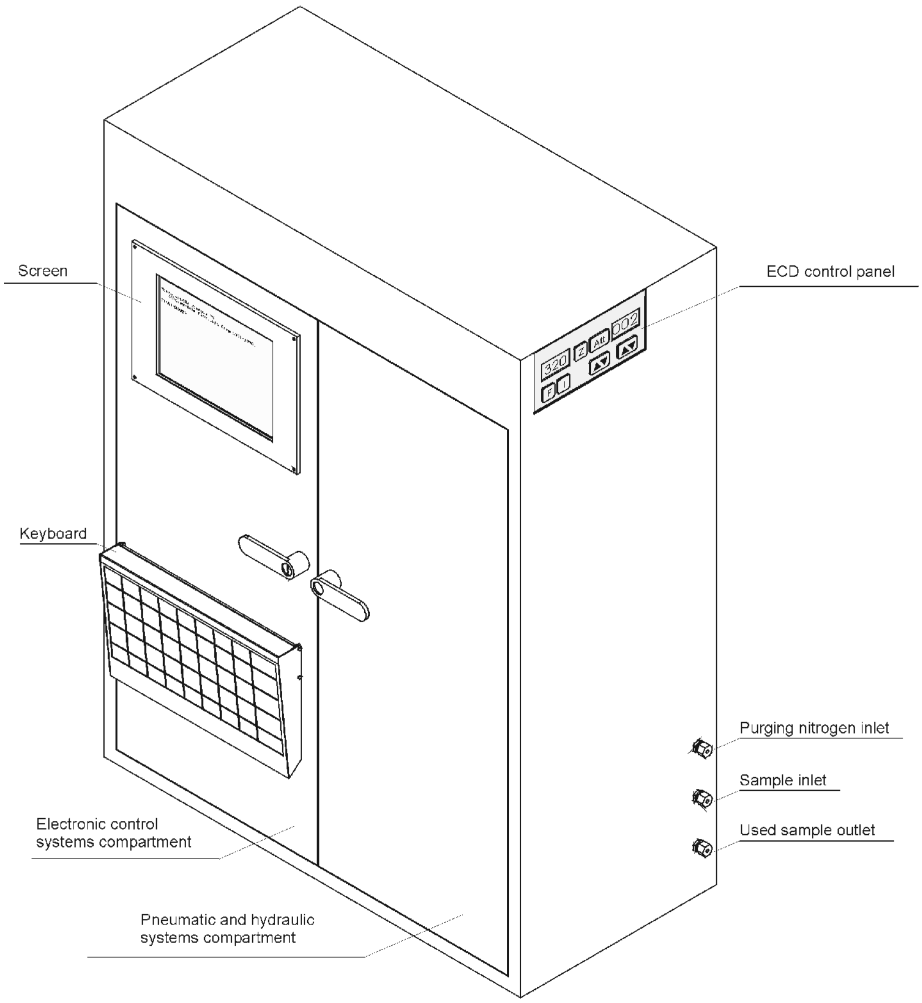

*Figure 4. External view of the instrument housing. A collapsible keyboard is shown in a rest position. Total weight of the instrument is ~25 kg (without the pump). Dimensions of the housing (height × width × depth) are: 900 × 700 × 300 mm. Total maximum power rating: 400 W.*

Pentium® III, 400 MHz microprocessor, 64 MB RAM, 1.2 GB HDD and 1.44 MB FDD (use of a mouse is optional). The PC operates under the Microsoft Windows '98™ operating system. The principal interface is based on a model LTI16V03 card (Latech, Puławy, Poland), including 16-channel, 12-bit ADC, two-channel, 12-bit DAC, 16 binary inputs and 16 binary outputs (TTL). Maximum operating frequency for the ADC is 100 kHz. An additional analogue circuit was constructed for interfacing the computer with control devices like temperature and pressure sensors, valves, heaters and fans. This circuit also generated a standard output for the transmission of analysis results to the main control room. Four signals are sent: a standard analogue current signal (4–20 mA), corresponding to the most recent concentration of chloro-organic compounds in analysed water, and three binary signals indicating (when set high).

- Concentration corresponding to 0–4 ppm range versus the normal 0–2 ppm range.
- Start-up or a calibration phase of instrument operation (when the analogue signal does not provide actual information) versus a regular analysis cycle.
- Instrument failure versus normal operation.

A suitable control program was written in MS Visual Basic language. It permits one to perform the following operations.

- Start the instrument.
- Calibrate the sensors of water sample level in the purging bubbler.
- Set the time interval for integration of the desorption peak.
- Perform the calibration procedure of the system.
- Start a normal operation cycle.
- Get information on key parameters of instrument operation, i.e. a detector signal that is also visualized on the screen, most recent analysis result (the one being transmitted via the analogue line), current value of the calibration coefficient (AMT/AREA), current temperature of both traps, temperature of the water sample, gas pressure, state of all TTL inputs and extensive information on instrument status including date + time of starting the automatic cycle, date + time of start of a particular phase of the cycle (stripping or desorption, indicating trap number) and, in a case of instrument failure, identification of its type and possible causes.

The program has an on-line help included. It was designed to facilitate the use of a keyboard, although mouse operation is also possible.

All the information delivered by the program is abundant when normal operation of the instrument is considered and is of importance only for personnel training and/or diagnostics. Other cases when the programs' capabilities are fully utilized are starting or calibrating the instrument and checking instrument failures. In the latter case, a suitable binary signal is sent to the control room and the details are shown on the screen, where the alerted serviceperson may check them. The following failures can be identified.

- Trap overheating (identifying trap no.).
- Too high a gas pressure.
- Thermocouple failure (indicating trap no.).
- Too high a sample level in the bubbler (impossible to maintain using normal control procedures).
- Too low a sample level in the bubbler.
- Analogue interface failure (its automatic reset by a watchdog circuit).

A program main window is shown in figure 5 and those items that are not self-evident are explained. Analogue-to-digital conversion is performed at 500 Hz and each of 50 measurements is averaged to produce a single point of the curve. After desorption is completed, the baseline is determined and the area between the curve and the baseline is integrated. In all operating modes but 'A' (figure 5), the results are displayed in area units, while in that mode the output is in ppm with the current calibration coefficient also given in the status window.

### Results

Extensive tests of the instrument were run for 2 months. The conclusions may be divided into several groups.

### *General conclusions*

The extended failure detection system was of rare use. It worked when tested, i.e. when the respective 'failures' were forced to occur, but otherwise no failure signals were received.

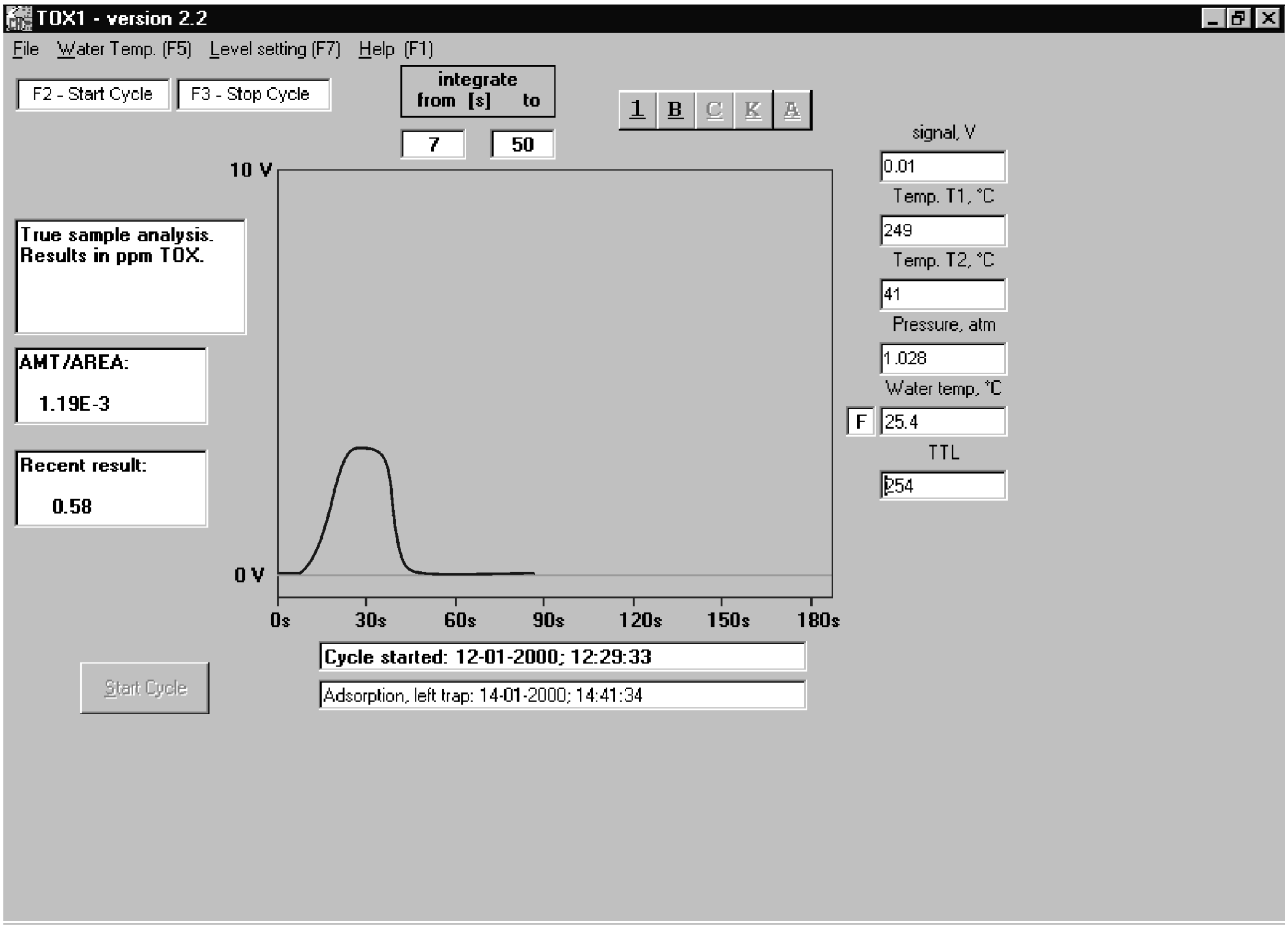

*Figure 5. Control program main window. 'F' at a water temperature reading means full control, i.e. signalling a failure when outside an interval allowed (the alternative is 'M', measurement only). Command buttons in the row marked 1, B, C, K and A serve for selecting operation mode: 1, running tests with AMT/AREA = 1, i.e. with results in units of area; B, measuring background signal (using 'zero' water); C, calibration, measuring standard solution 1; K, calibration, measuring standard solution 2, memorizing calibration curve; A, regular analysis.*

The concept of the 'dynamic' uncomplete purge, despite the requirement of maintaining a strict flow and temperature regime, was proven to be correct. As mentioned above, results are obtained once every 8 min, while it was found that a complete stripping required at least 24 min as one could obtain 'true zero' results (< 0.01 ppm) after at least three consecutive purges of the same portion of water. This system also permitted minimization of nitrogen consumption per analysis, which amounts to ~29 litres per 24 h, i.e. a typical cylinder (20 litres at 15 MPa) delivers gas for about 100 days of continuous operation.

Use of metal and/or fused silica tubing is necessary. When even small portions of plastic tubing were used, results were much more scattered, the phenomenon being unambiguously bound to the presence of volatile chloro-organic compounds in the air (even opening a bottle with dichloroethane for just few seconds in the vicinity raised the readouts). Nevertheless, the signal originates exclusively from organic chlorine (and, perhaps, other halogens) as it was found that obtaining 'zero' results was possible. The effectiveness of the utilized drying device was also proven in this way.

*Calibration and reproducibility*

The first problem faced was that of the background halogen content in water. After much experimentation, the following procedure was the most suitable.

- Freshly distilled and boiled (with continuous flow of pure nitrogen) water was cooled in a glass vessel used exclusively for these purposes and closed with a stopper equipped with a tube filled with active carbon to prevent any contamination from ambient air. Cooling was performed with nitrogen bubbling continued for 45 min. All handling of water was also performed in a way to prevent its contact with air. Dispensing water from the stock 5-litre vessel to the standard solution tank (1 litre) was performed without opening any of the two vessels, but by connecting them via tubing attached near to their bottoms (figure 6) and utilizing gravitational flow (the empty vessel had been filled with nitrogen prior to its filling). Even water prepared in this way yielded a signal corresponding to halogen contents of ~0.03–0.05 ppm. This value (as an area) had to be subtracted and the suitable step in the calibration procedure was developed and embodied in the program accordingly. Five measurements of background with 'zero' water were performed each time and the first of them, as markedly larger, was discarded. As mentioned above, the background was a 'true' one as after three subsequent purges of the same portion of water, the peak area was down to a value corresponding to concentration much < 0.01 ppm. It seems, however, that there is certain contribution originating from oxygen dissolved in water.

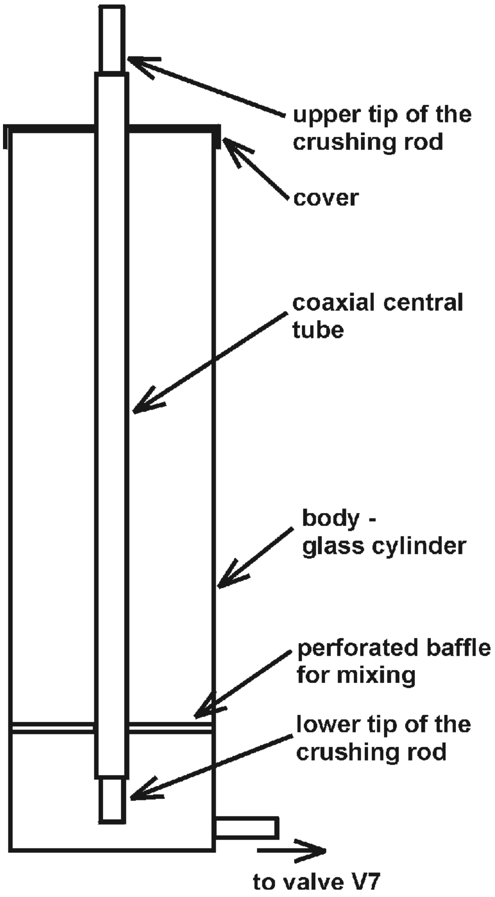

*Figure 6. Vertical cross-section of a vessel for preparation of standard solutions of dichloromethane and/or dichloroethane in water.*

- A special vessel (figure 6) had to be designed for preparation of calibration mixtures. It was a glass cylinder of 1 litre equipped with a cover and a central, coaxial tube reaching almost to the bottom. The vessel was filled with 'pure' water and a glass ampoule, containing a preweighted amount—8–100 mg—of a stock solution (~ 50 times more concentrated than the desired standard, containing either DIMA or one of its components in methanol) was inserted through the tube. Subsequently, the ampoule was crushed under water by means of a special rod introduced down the central tube. The tube had a perforated baffle attached for subsequent thorough mixing of the standard solution, which can be taken from the vessel via tubing attached at its bottom.
- Selected calibration curves (after background subtraction) are shown in figure 7. Three datasets are shown: triangles denote data collected with standard solutions containing dichloromethane (C1) only; squares, data collected with standard solutions containing dichloroethane (C2) only; and triangles collected with standard solutions containing DIMA (equimolar mixture C1 + C2). There are also regression lines drawn: a dotted line corresponds to dichloromethane, a dashed line to dichloroethane, and a solid line to both data sets collected. The line for the DIMA is for all practical purposes identical to the solid one. Parameters of the curves and corresponding coefficients of determination are shown in table 1. The data were collected over about 1 month. Results for C1 are slightly higher as apparently its lower Bp. (40 versus 57.5 °C) is reflected in its larger partition coefficient. This can also be seen in the shape of the thermal desorption curves, which are symmetrical for pure C1 or C2 standards, while for the mixture, the curve is widened at the descent side and exhibits stronger tailing (figure 5).
- The influence of the stripping gas flow rate on the result was tested by measuring the area for identical concentrations of each compound at flow rates differing by a factor of two. The results are shown in table 2. It is clear that even a huge change in gas flow leads to a minor variation in the response. Therefore, the influence of this factor may be neglected since its variations do not exceed 1–2 ml min$^{-1}$ (long-term) and the flow of the sample is maintained by the pump with both accuracy and precision being < 1% (relative). The results also prove that the sorbent bed size was chosen correctly, on the basis of earlier experience with a purge and trap technique, as a significant increase in a gas flow rate does not result in a breakthrough. It can also be concluded that the larger scatter of the C1 data must be caused by variations in stripping temperature rather than by any other reason. We tried to improve this situation by minimizing possible losses of the volatile C1 by using an additional 'floating cover' in the vessel shown in figure 6. However, no statistically significant reduction in C1 scatter was observed.
- All calibration curves were not linear. This was caused by somewhat difficult choice of the operating conditions of the ECD, which is a very sensitive detector, while the concentrations to be measured

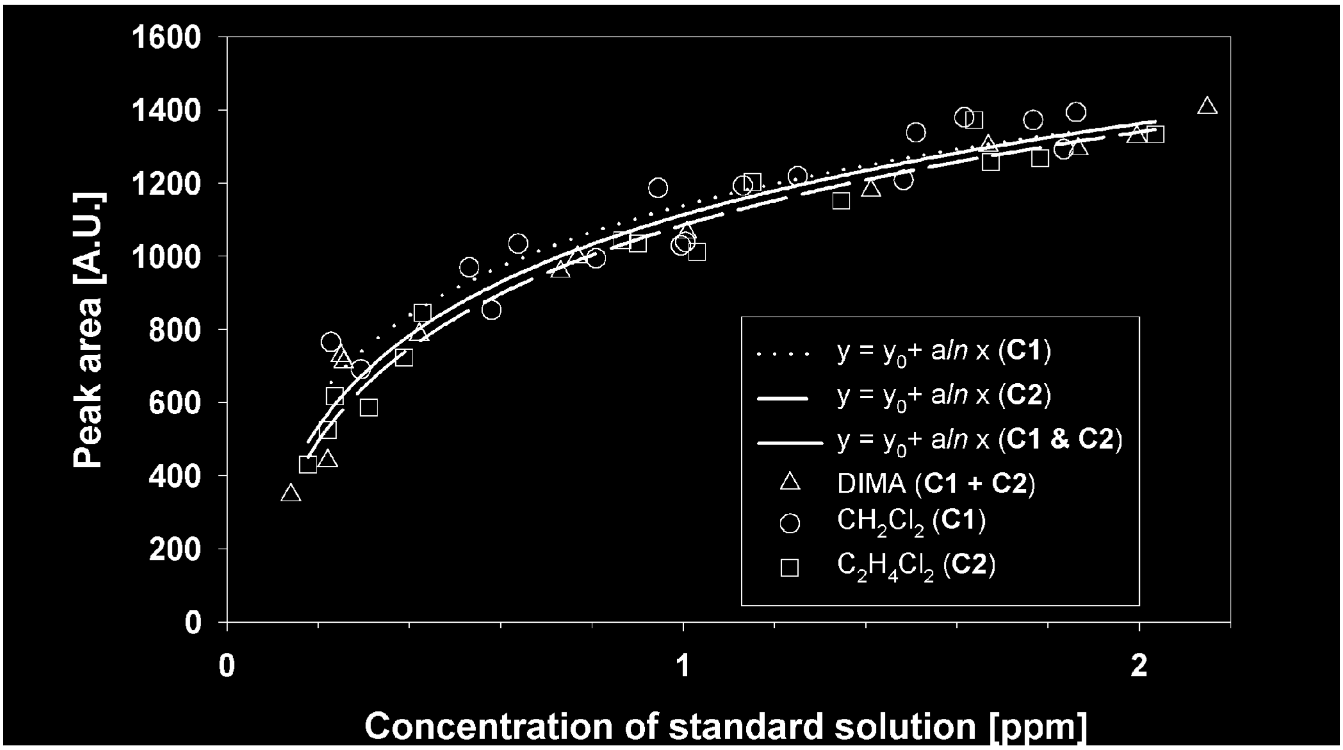

*Figure 7. Calibration curves: F, dichloromethane; G, dichloroethane; dotted line, regression for dichloromethane; dashed line, regression for dichloromethane; solid line, regression line for both datasets collected; △, DIMA (regression line for the latter is the same as the solid line; see text).*

*Table 1. Comparison of parameters of calibration curves obtained using different standard solutions. A row denoted (C1, C2) does not mean a separate standard solution, but parameters calculated on the basis of (C1) and (C2) datasets.*

| Standard solution | $y_0$ | $a$ | $r^2$ |
|---|---|---|---|
| $CH_2Cl_2$ (C1) | 1138 | 327 | 0.8896 |
| $C_2H_4Cl_2$ (C2) | 1086 | 368 | 0.9707 |
| (C1 and C2) | 1114 | 359 | 0.9325 |
| DIMA (C1 + C2) | 1093 | 353 | 0.9661 |

*Table 2. Comparison of calibration results obtained using different stripping gas flow rates. Concentrations of all solutions were identical and equal to* $1.01 \pm 0.017$ *mg Cl* $l^{-1}$.

| Standard solution | Gas flow rate (ml $min^{-1}$) | Peak area (a.u.) |
|---|---|---|
| $CH_2Cl_2$ (C1) | $23 \pm 1$ | $1035 \pm 5$ |
| $CH_2Cl_2$ (C1) | $46 \pm 1$ | $1117 \pm 3$ |
| $C_2H_4Cl_2$ (C2) | $23 \pm 1$ | $915 \pm 12$ |
| $C_2H_4Cl_2$ (C2) | $46 \pm 1$ | $945 \pm 13$ |

are relatively high [1]. Therefore, the operating conditions were chosen in such a manner as to lower the overall detector sensitivity: its operating temperature was 300 °C, reference current 1 nA, pulse amplitude 50 V and pulse width 1 ms. An alternative approach would mean operating the detector at a higher sensitivity with shortening of the stripping/adsorption time. This would lead to better linearity of the detector response, but at the same time to a much larger scatter, particularly with respect to C1. Therefore, it was decided to forget the linearity, although it was required to allow for a three-step calibration procedure (background + two standard solutions). Results $> 2$ ppm belong, in any case, already to the 'alert' zone when accuracy is not so important.

### Conclusions

A new type of a semicontinuous monitor for measuring levels of volatile chloro-organic compounds in industrial water, especially recirculated in cooling systems, has been designed and tested. It is based on a purge and trap technique. The first stage, i.e. that based on a continuous dynamic gas stripping, when gas and sample meet in a countercurrent bubbler with the respective flows is independently controlled. There are six independent variables that influence the effectiveness and recovery in this process (gas flow, sample flow, sample level in the bubbler, purging time, bubbler construction, temperature). The first four are controlled, the fifth is a constant, the sixth is controlled as far as the water intake (the bubbler itself is not thermostated). The trapping stage employs two Tenax TA® traps in parallel, which are used intermittently, thus ensuring that a result is obtained every 8 min, while a complete cycle (absorption–desorption–cooling) for a single trap lasts 13 min. Detection is ensured with an ECD Model 850 (CE Instruments) detector. The whole system requires a three-point calibration procedure before each operation cycle.

This computerized instrument ensures unattended operation over 2 months and possible faults are indicated automatically. So far, failures are rare (about one every 2–3 weeks) and all are correctly detected and identified by the system.

The concept of the 'dynamic' uncomplete purge was proven to be correct, permitting the shortening of the single analysis time (a complete 100% recovery stripping requires 24 min per sample under the existing conditions) and reducing the amount of nitrogen gas used per analysis. It has been found, however, that the scatter, observed in calibration curves, while still granting the desired precision and accuracy, might be reduced when a complete stripping (with only the gas flowing through the bubbler, while water sample volume remains constant after a refill with a fresh sample) was employed. This may be explained because any, even small, variations in two flows influence recovery, which depends on the *ratio* of the two flows. It is then suggested that a system may be used where stationary (gas flow only) stripping is employed, while recovery remains $< 100\%$ and is time controlled. This will be probably tested in a new model of the instrument.

No significant differences were found when the system was calibrated using either one of the two compounds (dichloromethane, Bp. 40 °C; dichloroethane, Bp. 57.5 °C) or both. No chromatographic effects (separation) on the sorbent bed that might influence the quantitative results were observed.

### References

1. Poole, C. F. and Schuette, S. A., 1984, *Contemporary Practice of Chromatography* (Amsterdam: Elsevier), pp. 170–181 (ECD), 469–472 (purge and trap).
2. Wasik, A., Janicki, W., Wardencki, W. and Namieśnik, J., 1997, *Analusis*, **25**, 59.
3. Zygmunt, B., 1996, *Journal of Chromatography A*, **725**, 157.
4. Zygmunt, B., 1997, *Journal of High Resolution Chromatography*, **20**, 482.
5. Namieśnik, J., Górecki, T., Biziuk, M. and Torres, L., 1990, *Anal. Chimica Acta*, **237**, 1.
6. Hovorka, S. and Dohnal, V., 1997, *Journal of Chemical Engineering Data*, **42**, 924.
7. Namieśnik, J., 1988, *Chemical Analysis*, **33**, 835.

